# Macrofossil evidence unveiling evolution of male cones in Ephedraceae (Gnetidae)

**DOI:** 10.1186/s12862-018-1243-9

**Published:** 2018-08-29

**Authors:** Yong Yang, Longbiao Lin, David K. Ferguson, Yingwei Wang

**Affiliations:** 1 State Key Laboratory of Systematic and Evolutionary Botany, Institute of Botany, Chinese Academy of Sciences, 20 Nanxincun, Xiangshan, Beijing, 100093 China; 2 China Railway Group Limited, 69 Fuxing Road, Beijing, 100039 China; 3 Department of Paleontology, University of Vienna, 1090 Vienna, Austria; 4 Beijing Botanical Garden, Chinese Academy of Sciences, 20 Nanxincun, Xiangshan, Beijing, 100093 China

**Keywords:** Early cretaceous, Ephedra, Evolution, Gnetophytes, Jehol biota, Male cone, Yixian formation

## Abstract

**Background:**

Male cones of modern Ephedraceae are compound and compact. No fossil evidence has so far been found to support an origin of the compact compound male cone from a hypothetical loosely-arranged shoot system.

**Results:**

Here we describe a new macrofossil taxon, *Eamesia chinensis* Yang, Lin, Ferguson et Wang, gen. et sp. nov., from the Early Cretaceous of western Liaoning, northeastern China. It was an ephedroid shrub bearing male spikes terminal to twigs, but differs from modern Ephedraceae by its loosely-arranged male cones, the axillary male shoot consisting of an elongated synangiophore on which leaf-like foliar organs were inserted, and four sessile synangia terminal to the apex.

**Conclusions:**

The morphology of this fossil suggests that the modern compact male cone of Ephedra was indeed derived from a once loosely-arranged shoot system, and the male reproductive unit originated from a once elongated axillary male shoot. This new fossil species thus provides a transitional link from the hypothetical ancestral shoot system to the modern compact morphology. Changes of habitat from closed humid forests to open dry deserts and shifts of the pollination syndrome may have acted as the driving forces behind this morphological evolution.

## Background

The gnetophytes contain three extant monotypic families, i.e. Ephedraceae (*Ephedra* L.), Gnetaceae (*Gnetum* L.), and Welwitschiaceae (*Welwitschia* Hook. f.); these live in different habitats and possess divergent morphology [1]. The Ephedraceae are common in cold and dry places in both the Old- and the New Worlds, the Gnetaceae live in warm and humid tropical/subtropical forests of Asia, Africa, and South America, while the Welwitschiaceae only occur in warm and dry coastal places in southwestern Africa [1]. The Ephedraceae are shrubs, rarely small trees or lianas, the Gnetaceae are usually lianas, rarely small trees, while the Welwitschiaceae are represented by a single species, *Welwitschia mirabilis,* which is a dwarf shrub [1]. The Ephedraceae are basal within the living gnetophytes [2, 3], but possess extremely reduced morphology compared to the Gnetaceae and Welwitschiaceae, e.g. small linear leaves with simple parallel veins (vs. broad leaves with pinnate venation in the Gnetaceae, and giant leaves with multiple parallel veins having interconnected interveins in the Welwitschiaceae), reduced female cones with only the uppermost pair/whorl of bracts fertile (vs. multiple pairs/whorls of fertile bracts in the Gnetaceae and the Welwitschiaceae), usually unisexual male cones (vs. usually bisexual male cones having abortive enveloped seeds in the Gnetaceae and the Welwitschiaceae) [1]. These morphological characters of the three families are so different from each other that classifying them as a monophyletic group was sometimes questioned. Based on an evolutionary analysis of cone morphology, Eames [4] concluded that the Ephedrales are descendants of the Cordaitales, while the Gnetales and Welwitschiales are close to cycads. Meyen [5] laid emphasis on seed symmetry, and assigned the Ephedrales to the Ginkgoopsida, while placing the Gnetales and Welwitschiales in the Cycadopsida. Subsequent cladistic studies either suggested that the gnetophytes comprise a monophyletic group [6], or a paraphyletic group [7]. However, modern molecular phylogenetic studies strongly support them as belonging to a single clade [2, 3]. The partially enveloped seeds and the presence of foraminate vessels can unite the three families/orders into a monophyletic group [8]. It remains a riddle as to how these plants evolved from the common ancestor considering their huge morphological gaps.

A large number of macrofossils showing gnetalean morphology has been reported from the Mesozoic since the end of the last century, e.g. in Asia [9–26], in Australia [27], in Europe & North America [28–32], and in South America [33–38]. Some of them were described as angiosperms, e.g. *Chaoyangia* Duan≡*Gurvanella* Krassilov, *Liaoxia* Cao et Wu, *Callianthus* Wang et Zheng≡*Erenia* Krassilov, *Baicarpus* Han et al., and *Pseudoephedra* Liu et Wang, but actually belonged to the gnetophytes [21, 22, 39]. These macrofossils were mostly female [15–17, 20, 24–26], and rarely male (e.g. *Khitania* Guo et al. of the Gnetaceae [10]; *Welwitschiostrobus* Dilcher et al. of the Welwitschiaceae [34]), or bisexual (e.g. *Callianthus dilae* Wang et Zheng [38]) or monoecious (e.g. *Friedsellowia* Löwe et al. [35]). These macrofossils have greatly enhanced our understanding of the early evolution of the female cones of gnetalean plants, especially those ephedroid fossils from northeastern China [16, 17, 20–26], and a reduction-sterilization model integrating morphological, anatomical, ontogenetic, and palaeobotanical evidence has been developed to explain the origin and evolution of the modern reduced and compact female cone of the Ephedraceae [21–25]. However, thus far little evidence has been found to unveil the evolution of male cones in the gnetophytes.

**Fig. 1:**
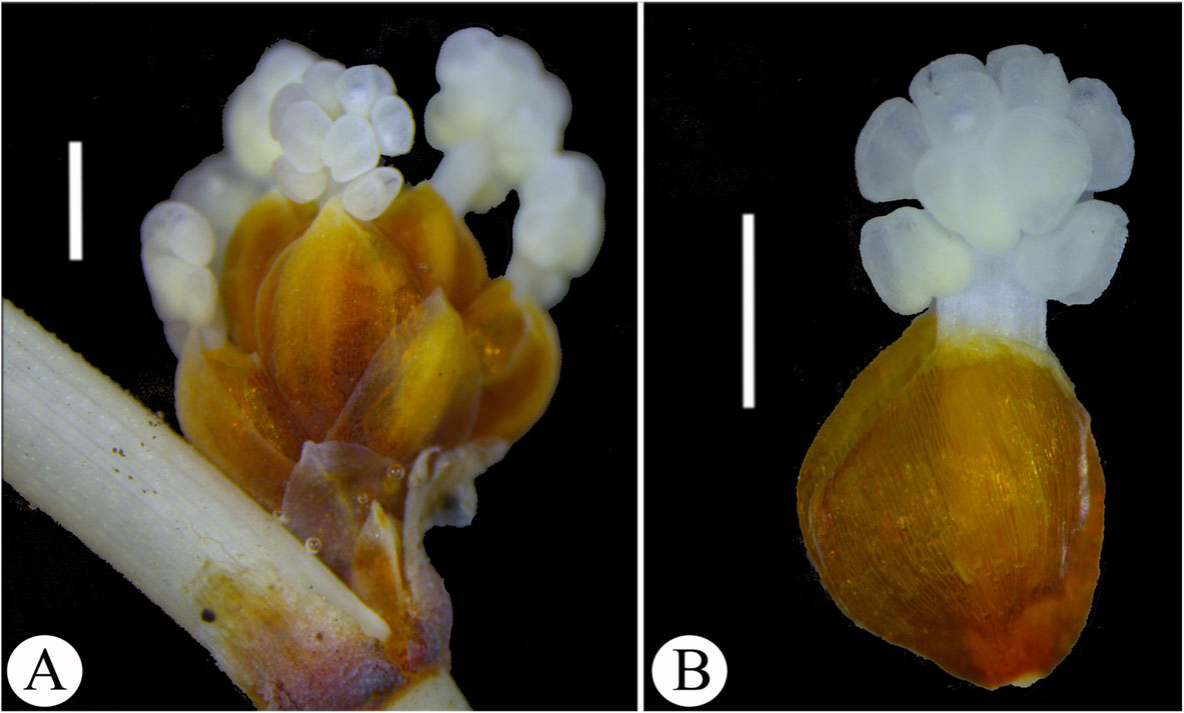
Morphology and structure of male cones of *Ephedra intermedia*. **a**, a male cone; **b**, a male reproductive unit

Male reproductive organs of the gnetophytes are markedly diversified. Male cones of *Ephedra* are compound (Fig. 1), consisting of two to 13 whorls of binately or ternately arranged bracts, each bract excepting the lowermost pair subtends an axillary male reproductive unit which has a pair of dorso-ventrally fused bracteoles enclosing a middle synangiophore with 3–12 apical sessile to stipitate synangia [40, 41]. However, a few species sometimes bear aborted female reproductive units which are enclosed in the uppermost pair of bracts [2, 41, 42]. Occasionally bisexual reproductive units occur in a male cone [43]. Male spikes of *Gnetum* usually possess aborted female reproductive units above the male whorls [42, 44], while *Welwitschia* normally has male cones consisting of ‘bisexual’ reproductive units, each of which includes six basally fused synangiophores and a central aborted ovule with a long micropylar tube [1].

Though Eames [4] and Mundry & Stützel [45] both support a close relationship between the Ephedraceae and the Cordaitales, their explanations on evolution of the male cones of the Ephedraceae are fundamentally different. Eames [4] hypothesized that male cones of *Ephedra* are compound, early cones are comparable to those of *Cordaianthus*, the axillary male reproductive unit is a modified reproductive shoot (‘simple strobilus’), and the pair of fused bracteoles was derived from leaves, while the middle synangiophore represents a pair of fused microsporophylls. Mundry & Stützel [45] proposed a much more complicated model, in which the hypothetical ancestor of the gnetophytes was considered to be bisexual, its reproductive shoot system branched at least twice and is thus super-compound with an apical compound female cone and several pairs of lateral compound male cones. In this scenario, the modern synangiophore was derived from a male cone by reduction of foliar organs and shortening of the sporophyll-bearing secondary shoots, the modern synangiophore being formed by the fusion of two reproductive shoots, with each shoot having four terminal synangia. The modern axillary male reproductive unit is thus a compound cone. Despite their difference, both models suggest that the male cone of the Ephedraceae was derived from a loosely arranged shoot system, but no direct fossil evidence has been found to support this thus far.

There are a few fossil gnetalean male cones reported from the Early Cretaceous, but all of them are specialized and no structural details were preserved: *Khitania* is close to *Gnetum* [10], *Welwitschiostrobus* belongs to the Welwitschiaceae [34], while *Friedsellowia* possesses an ambiguous affinity to living families of Gnetidae [35]. Although important finds, these plant fossils are not helpful for testing the competing hypotheses on the evolution of male cones in the Ephedraceae.

The Early Cretaceous Yixian Formation of northeastern China is well known for its excellent fossil preservations including dinosaurs, early birds, and the early angiosperm Archaefructaceae [18, 46, 47]. The Yixian Formation consists of fossiliferous lacustrine sediments, with a plant fossil assemblage which indicates that the palaeoenvironment was once warm and humid, with a subtropical flora dominated by conifers [48]. Frequent volcanic eruptions, together with forest fires and poisonous gases resulted in frequent destructions/reestablishment of the local vegetation, and thus affected the evolution and diversification of both plants and animals [46]. Here we report a new ephedroid macrofossil from the Early Cretaceous of northeastern China which is significant for understanding reproductive evolution in the Ephedraceae.

## Results

### Gymnosperms

**Subclass** – Gnetidae Pax.

**Order** – Ephedrales Dumortier.

**Family** – Ephedraceae Dumortier, emend [21].

**Fig. 2:**
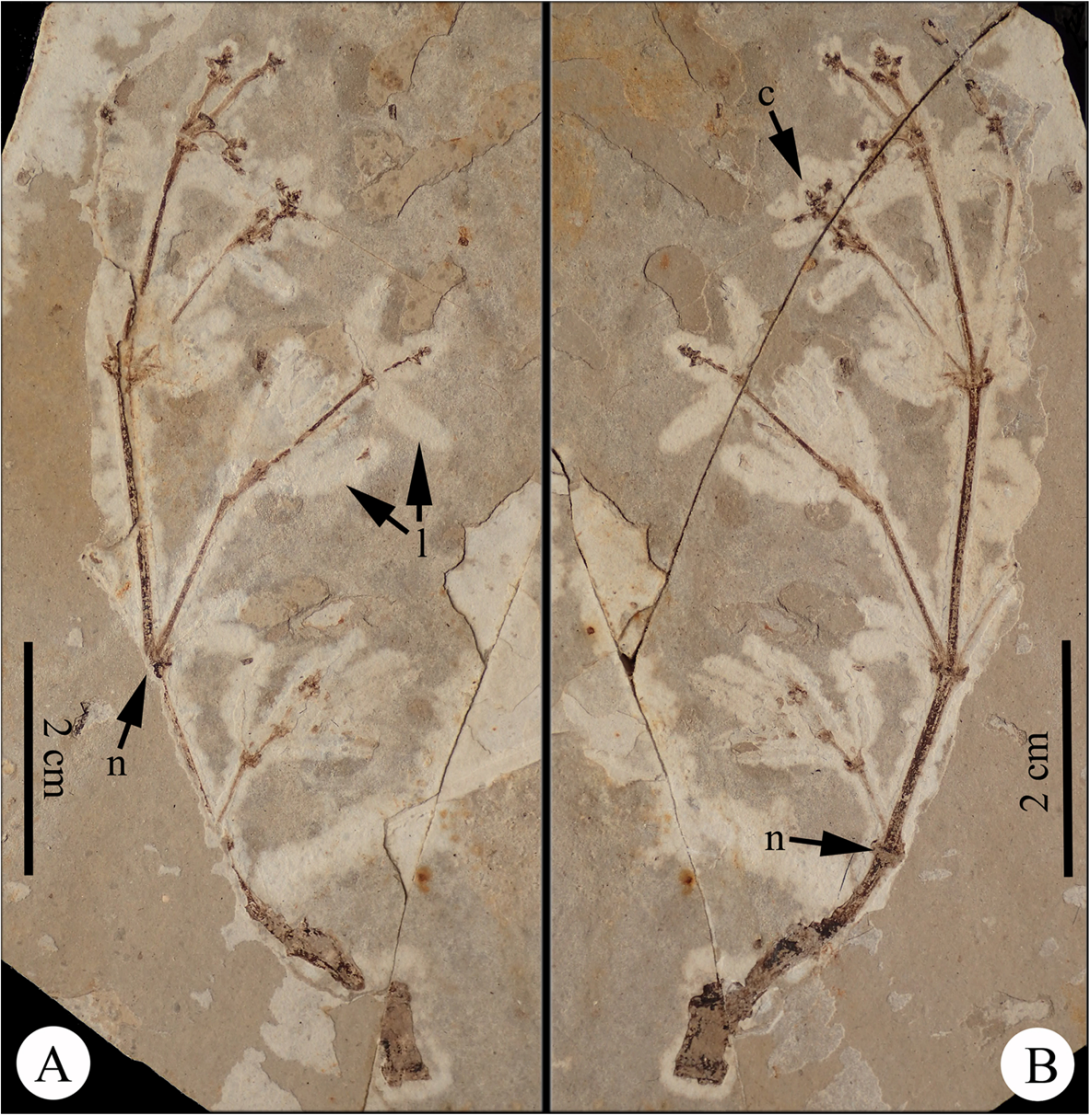
Part and counterpart of *Eamesia chinensis*. **a**, part; **b**, counterpart. *Abbreviations*: **c**, male cone; l, leaves; n, node

**Fig. 3:**
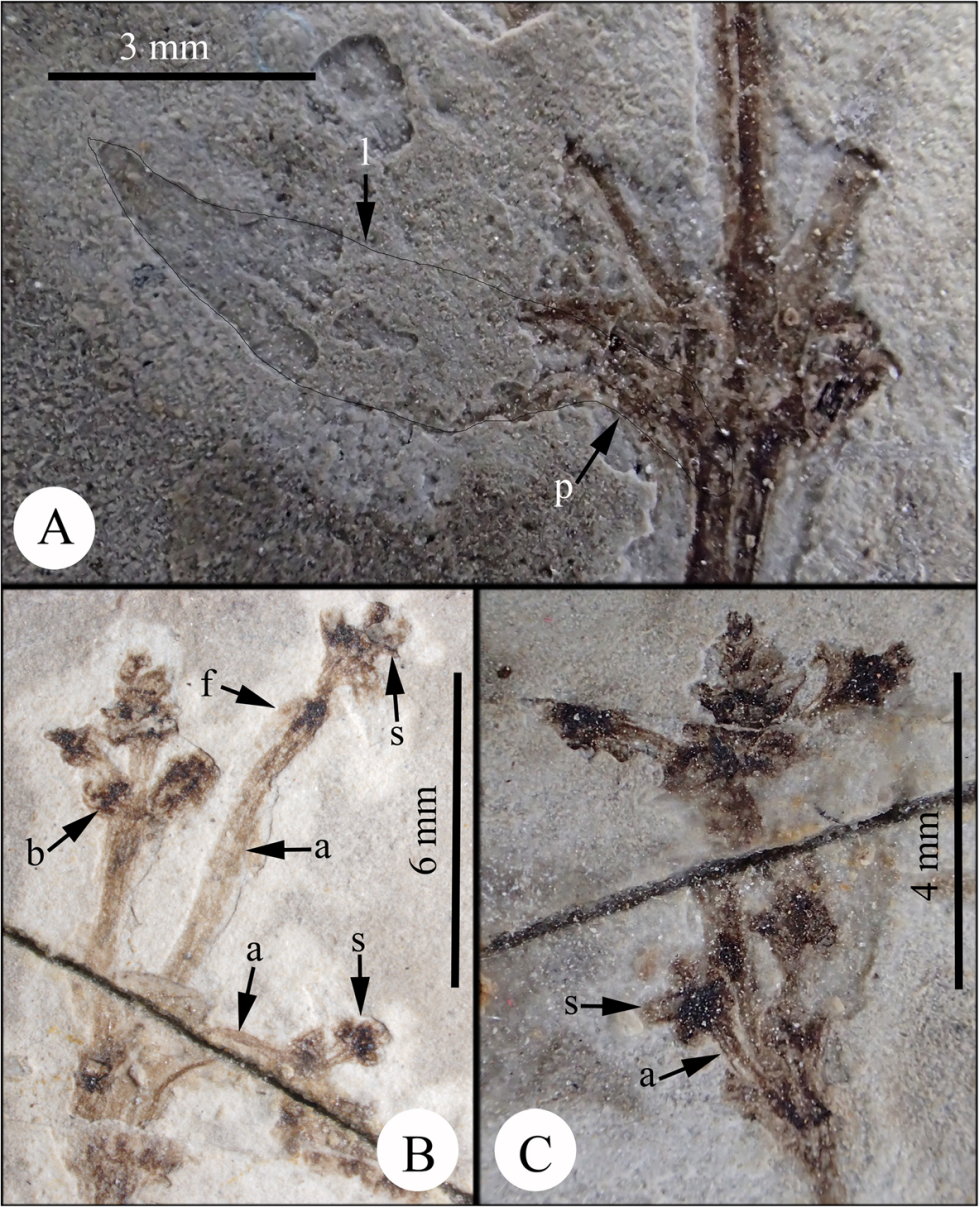
Morphology of *Eamesia chinensis*. **a**, nodal portion magnified showing the linear to lanceolate leaf shape; **b** and **c**, male cone magnified showing the scale-like bracts, the elongated synangiophore, the sessile synangia, and the linear foliar organ. *Abbreviations*: a, synangiophore; b, bract; f, foliar organ; l, leaf; n, node; s, synangia; v, vein

**Fig. 4:**
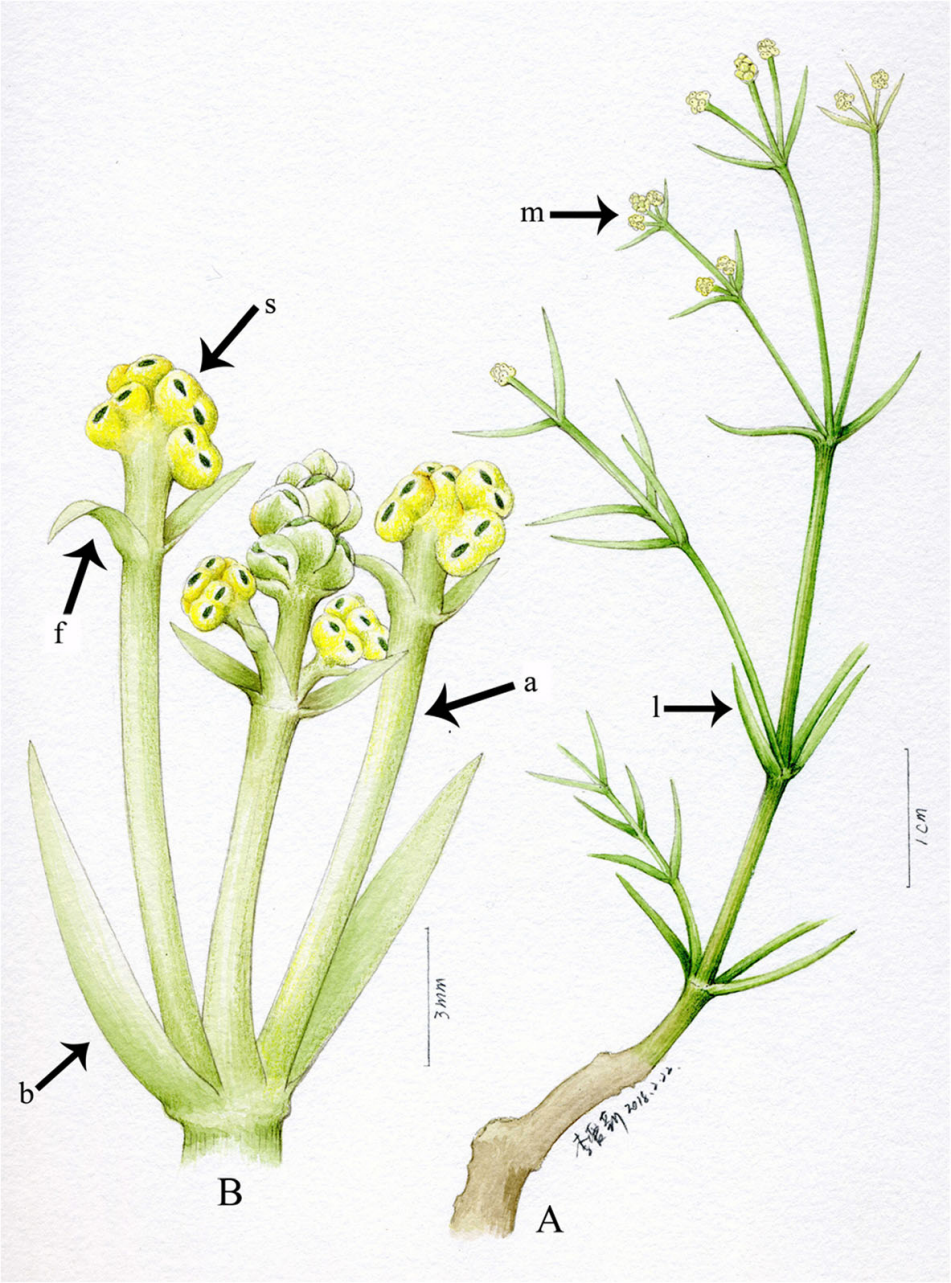
Reconstruction of *Eamesia chinensis*. **a**, plant portion displaying the habit, the decussate phyllotaxy, and the terminal male cones; **b**, male cone magnified displaying the loosely organized male spikes, the acropetal developmental pattern, and the modified synangiophore having a pair of foliar organs below the terminal synangia. *Abbreviations*: a, synangiophore; b, bract; f, foliar organ; l, leaf; m, male spike; s, synangium

**Species** – **Eamesia chinensis** Y. Yang, L.B. Lin, D.K. Ferguson et Y.W. Wang, gen. et sp. nov. Figs. 2, 3 and 4.

**Diagnosis** – The plant is ephedroid; shoot dichasially branched; nodes swollen; internodes longitudinally striated. Male cones terminal on twigs, laxly arranged; synangiophore elongated, and possesses four sessile synangia apically, below the synangia are a (pair of) free linear foliar organs.

**Description** – The preserved plant consists of part and counterpart (Fig. 2a & b). Plant ca. 9.1 cm tall, possessing swollen nodes and finely striated internodes, dichasially branched at nodes. Shoot ca. 4 mm thick at the base, tapering distally. Leaves opposite at nodes, ascending to spreading, linear to somewhat lanceolate, 7–9 mm long, 1–2 mm broad, at base slightly narrowed into a short petiole; leaf veins parallel (Fig. 3a). Male cones terminal on twigs, 4–10 mm long, possessing at least three pairs of bracts, developed acropetally, laxly arranged; the lowermost internode up to 6 mm long (Fig. 3b & c). Cone bracts linear and leaf-like, each subtending a synangiophore, 3–10 mm long; four sessile synangia distal to each synangiophore (Fig. 3c); a (pair of) linear foliar organ(s) present below the synangia (Fig. 3b). The synangia probably had apical pores.

**Etymology** – The generic name “*Eamesia*” is given in honour of the plant morphologist Arthur J. Eames, while the specific epithet “*chinensis*” is named for China where the type originates from.

**Holotype** – 2015092901A & B (Fig. 2a & b) (Part and counterpart specimens of a single collection, here designated).

**Repository** – Chinese National Herbarium (PE), Institute of Botany, Chinese Academy of Sciences, Beijing, China.

**Fig. 5:**
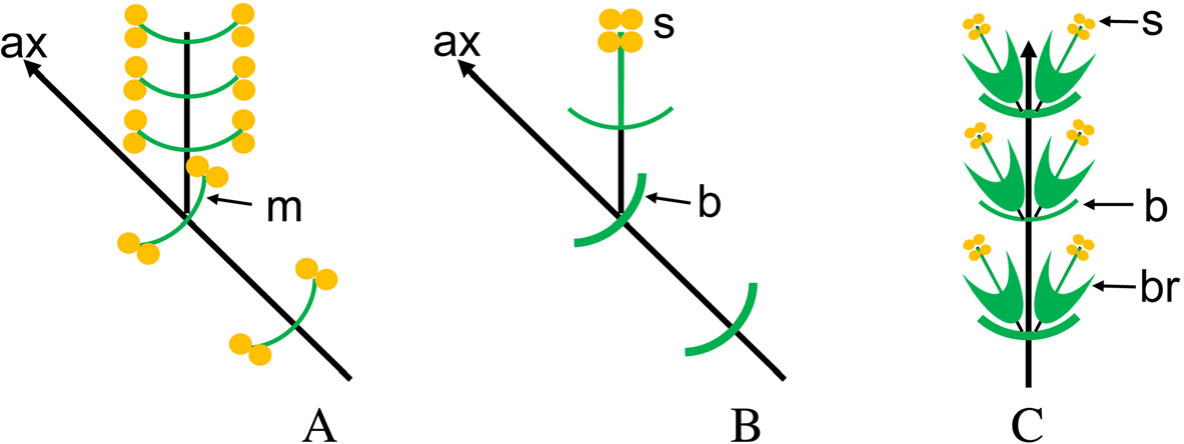
Illustrations displaying evolution of the modern compact cones from ancestral male shoots through transitional lax male spikes. **a**, the hypothetical ancestral male shoot; **b**, the transitional lax male spikes of *Eamesia chinensis*; **c**, the modern compact male cone of modern Ephedraceae. *Abbreviations*: ax, axis of reproductive shoots/male cones; b, bracts/foliar organs on primary axis of male cones/spikes; br, bracteoles on the secondary axis of male cones/spikes; m, microsporophylls; s, synangium

**Type locality** – Dawangzhangzi Village, Songzhangzi Town, Lingyuan City, Chaoyang District, Liaoning Province, China (Fig. 5).

**Stratigraphic horizon and age** – Early Cretaceous, Daxinfangzi Bed (previously known as Dawangzhangzi Bed), Yixian Formation (Early Aptian-earliest Late Aptian of the Early Cretaceous).

**Remarks** – *Eamesia chinensis* Yang, Lin, Ferguson et Wang is the first ephedroid plant from the Early Cretaceous displaying structural details of the male cones. The new plant differs from modern *Ephedra* in its laxly arranged male cones having an elongated axillary synangiophore (or male shoot) with linear foliar organs in the upper portion just below the sessile synangia (vs. compact male cones bearing an axillary sessile male unit consisting of a pair of bracteoles enclosing an inner synangiophore in *Ephedra*). The male cones developed in an acropetal manner, the proximal synangiophores were elongated, while the distal ones became shorter and were under-developed. Synangia of the lower pair of male shoots were well-developed, indicating that this plant was in the pollination phase. We did not recognize any female units in this plant, and believe that this is a unisexual male plant.

This plant was probably a shrub, because the stem was thick and seems woody proximally, becoming attenuated distally. There are linear white marks opposite at most nodes; being so regular, we believe these must represent leaf remains. We did not notice any such leaf remains in many other ephedroid macrofossils from the Yixian Formation. The leaf remains of *Eamesia chinensis* were probably tender and slightly fleshy. Based on our morphological study, we reconstructed the plant in a line drawing (Fig. 4).

## Discussion

### Comparisons with other known gnetophytes from the early cretaceous

Fossil gnetophytes have a high morphological diversity and wide distribution. Fossils of male plants related to the Gnetaceae are rare, only represented by *Khitania* from the Early Cretaceous of northeastern China [10]. The external morphology of *Khitania* is very close to that of modern *Gnetum*. It consists of a bifurcate reproductive shoot with two apical elongated male cones with many whorls of annular involucral bracts, but no structural details are preserved [10]. *Eamesia chinensis* Yang, Lin, Lin, Ferguson et Wang differs markedly from *Khitania* by its laxly arranged male cones with opposite and decussate reproductive units (vs. compact cones with annular bract collars).

The Welwitschiaceae were distributed in southwestern Europe, eastern North America, and northern South America in the Early Cretaceous [28, 30, 34]. Fossils from the Northern Hemisphere are either seed mesofossils (*Becatia* Friis et al.) [30] or female plants (*Drewria* Crane et Upchurch) [28]. *Eamesia chinensis* is similar to *Drewria* in its dichasial branching pattern and the opposite linear leaves, but differs in possessing smaller leaves (7–9 × 1–2 mm vs. 10–20 × 2–6 mm in *Drewria*). *Welwitschiostrobus* is a male cone from South America [34]. *Eamesia chinensis* is similar to *Welwitschiostrobus* in the opposite reproductive units, but differs from the latter in its laxly arranged cones (vs. compact cones). Löwe et al. [35] reported a monoecious plant from the Lower Cretaceous Crato Formation of South America: *Friedsellowia* Löwe et al., which is close to *Welwitschia*. It differs from our new species in its giant and monoecious habit, and the tightly arranged male cones subtending a central female cone.

Many ephedroid macrofossils from the Early Cretaceous of northeastern China and Mongolia have been found, some of which are preserved with reproductive shoots [13–26]. These macrofossils display similarities to our new species including a dichasial branching pattern, articulate branches having swollen nodes and longitudinally furrowed internodes, as well as opposite and decussate leaves (if present; leaves are not seen in *Ephedra hongtaoi* Wang et Zheng, and *E. archaeorhytidosperma* Yang et al.). Leaves of *Eamesia chinensis* are linear and simple, as in *Gurvanella* Krassilov (syn.: *Chaoyangia* Duan et al.) [49, 50], *Liaoxia* Cao et Wu [17], *Prognetella* Krassilov [22], *Chengia* Yang et al. [25], and *Siphonospermum* Rydin et Friis [16]. They differ from *Spinobractea lanceolata* Liu et al. and *Constrobilus ovata* Liu et al. by their smaller linear leaves 7–9 mm long (vs. larger ovate broad leaves 20–35 mm long [14]), from *Latibractea divisa* Liu et al. by the simple leaves (vs. larger divided leaves in the latter species [14]), and from *Ephedra multinervia* Yang et al. by the smaller linear leaves less than 1 cm (vs. larger strap-like leaves ca. 4.9 cm long [24]).

*Eamesia chinensis* also shows similarities to a few female macrofossil gnetalean species from South America in the dichasial branching pattern and opposite leaf arrangement, e.g. *Itajuba yansanae* Ricardi-Branco et al. [37], *Cariria orbiculiconiformis* Kunzmann et al. [51], *Ephedra verticillata* Cladera et al. [33].

Unfortunately, it is not possible to compare *Eamesia chinensis* with a few other ephedroid species preserved only as seed mesofossils [29, 32] or female cones, e.g. *Pseudoephedra paradoxa* [15], *Ephedra carnosa* Yang et Wang [26], and *Protoephedrites* Rothwell et Stockey [31]. *Erenia* (syn.: *Callianthus* Wang et Zheng) is a female cone [21, 52], although Wang and Zheng [39] thought it was a bisexual “flower” due to their mistaken interpretation of the fragmentary bracts as male structures.

In its vegetative morphology and paired axillary male reproductive units, *Eamesia chinensis* shows the closest similarity to *Ephedra* within the living gnetophytes. It differs from *Gnetum* by the cone lacking annular collars and leaf morphology (linear and parallel-veined vs. pinnately veined broad leaves in *Gnetum*), and from *Welwitschia* by the lack of bisexual reproductive units in male cones, the dwarf habit, and the giant leaves.

### Shortening of synangiophore and evolution of bracteoles

Previous botanists suggested that the synangiophore is a compound structure derived from fusion of two microsporophylls because the synangiophore is sometimes bilobed in *E. intermedia*, *E. distachya* and *E. nebrodensis* (≡*E. major*) [1, 4, 53]. Such lobed synangiophores are not observed in *Eamsia chinensis*, in fact, the synangiophore of this fossil species is completely united and the four synangia are all sessile. As a result, our new species does not help in resolving this hypothesis. However, the new fossil species does provide evolutionary clues in other aspects.

Firstly, the male cone of the Ephedraceae was considered to be compound, implying that the axillary male unit was derived from a secondary shoot. There is no other evidence to support the shoot nature of the male unit except for its axillary position. In the fossil species, the male unit is elongated and displays shoot features, for instance, the male unit is axillary to a bract and possesses a long proximal stalk below the foliar organs that correspond to the bracteoles of modern species. This may confirm the male shoot hypothesis.

Secondly, male cones of modern Ephedraceae are compound and compact. Our macrofossil *Eamsia chinensis* possesses loosely arranged male spikes having both elongated syangiophores and internodes on the cone axis, thus possessing transitional morphology linking a hypothetical ancestral shoot system of male shoots to the modern compact compound male cones [4, 45]. This evolutionary trend is illustrated in Fig. 5. In addition, the foliar organs on the synangiophore are leaf-like, while they show highly specialized morphology in modern species. Thus, *Eamesia chinensis* provides the first unequivocal palaeobotanical evidence linking modern Ephedraceae to its hypothetical ancestor. This evolutionary trend was also observed in female cones of the gnetophytes [22]. The evolutionary change from a loosely arranged male shoot/spike to a compact male cone may be related to a shift in the living environment. Early Cretaceous ephedroid plants including *Eamesia chinensis* lived in humid forests and close to rivers/lakes as in the case of *Prognetella* [22], *Chengia* [25] and *Ephedra hongtaoi* [20], whereas the modern species occur in open dry deserts. We hypothesize that the compact male cones probably function more effectively than loose spikes in open environments under windy conditions. Modern *Ephedra* usually lives in open, dry and windy areas, is a subshrub with reduced and compact cones, whereas its relative *Gnetum* lives in tropical and subtropical forests, has an elongated habit (lianas or small trees) and lax spikes.

Basal species of Ephedraceae possess bisexual male cones and are entomophilous whereas the core group has unisexual male cones and are anemophilous. The shift of the pollination systems is thought to have taken place around the K/Pg boundary, possibly accompanied by evolution of pollen with pseudosulci [54]. Our new fossil species *Eamesia chinensis* from the Early Cretaceous possesses unisexual male cones; there is no sign of female units in the male spikes. It implies that the shift of pollination systems in Ephedraceae may have started in the Early Cretaceous.

## Conclusions

*Eamesia chinensis* Yang, Lin, Ferguson et Wang, the first ephedroid male plant consisting of a reproductive shoot bearing male cones, is described as new to science. This new macrofossil species from the Early Cretaceous Jehol Biota possesses ephedroid vegetative morphology, and laxly arranged male cones with elongated internodes on the cone axis and axillary male reproductive units. The morphology of *Eamesia chinensis* provides direct fossil evidence for an earlier hypothesis proposing a close relationship between Cordaitales and modern Ephedraceae.

## Methods

**Fig. 6:**
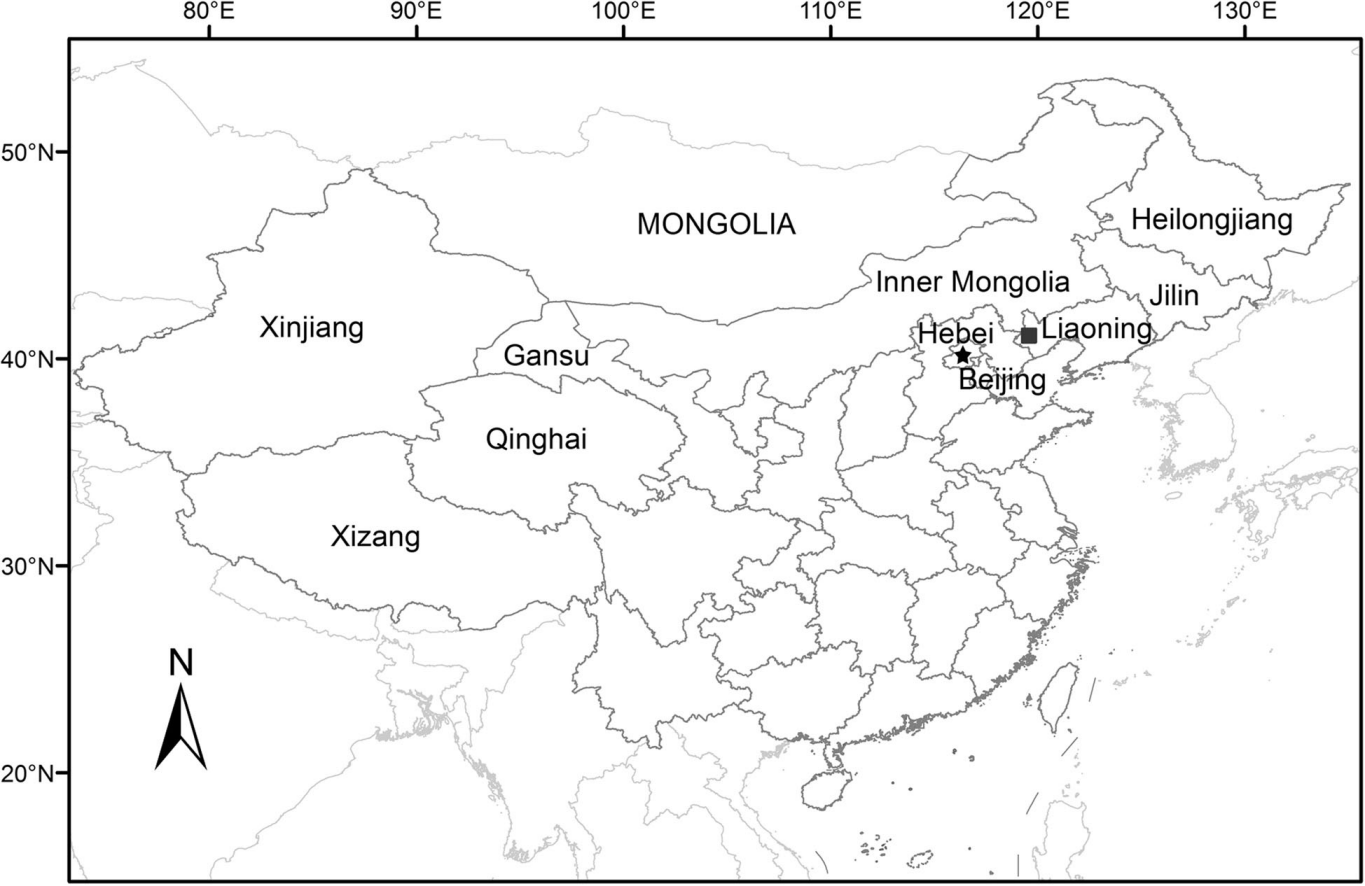
Type locality of *Eamesia chinensis* indicated by the black solid square (■). The map was generated using ArcGIS 9.3 (ESRI, Redlands, CA, USA; http://www.esri.com)

The plant fossil specimens are preserved as impressions, which were collected at Dawangzhangzi Village, Lingyuan City, Liaoning Province, northeastern China (Fig. 6). The lacustrine stratum containing the fossil specimens belongs to the Daxinfangzi Bed in the lower part of the Yixian Formation (previously known as Dawangzhangzi Bed) [55]. A few well-known angiosperm macrofossils were found in the formation, e.g. *Leefructus mirus* Sun et al., *Hyrcantha decussata* (Leng et Friis) Dilcher et al., and *Archaefructus sinensis* Sun et al. [55–57] and other diverse plant groups (ginkgophytes, conifers, cycadophytes, pteridosperms). Radiometric dating indicates that the Daxinfangzi Bed is about 122.6–125.8 Myr old [58–61]. This age corresponds to the Early Aptian - earliest Late Aptian of the Early Cretaceous in the International Stratigraphic Chart [62, 63].

The fossil specimens were photographed with digital cameras (Olympus TG-3) and under a microscope (Nikon Eclipse E600). Images were cut and edited with Photoshop CS2 ver. 9.0. The hand-drawn reconstruction of the fossil plant was made using colored pencils, and then edited using Photoshop CS2 ver. 9.0. The map of the type locality was generated using ArcGIS 9.3 (ESRI, Redlands, CA, USA; http://www.esri.com).

## Abbreviations

a: synangiophore
b: bract
c: male cone
f: foliar organ
l: leaf
m: male spike
n: node
s: synangia
v: vein
